# Environmental DNA-Based Identification of Non-Native Fish in Beijing: Diversity, Geographical Distribution, and Interactions with Native Taxa

**DOI:** 10.3390/ani14172532

**Published:** 2024-08-31

**Authors:** Bo Liu, Fuwen Wang, Shiguo Li, Wei Xiong, Aibin Zhan

**Affiliations:** 1 Beijing Hydrology Center, Beijing 100089, China; mak4@163.com; 2 Research Center for Eco-Environmental Sciences, Chinese Academy of Sciences, Beijing 100085, China; wangfuwenouc@163.com (F.W.); sgli@rcees.ac.cn (S.L.); 3 University of Chinese Academy of Sciences, Chinese Academy of Sciences, Beijing 100049, China

**Keywords:** eDNA, biological invasion, fish, non-native species, species interaction

## Abstract

**Simple Summary:**

We employed environmental DNA (eDNA) metabarcoding to assess fish biodiversity in three river basins in Beijing. Across all the 67 sampling sites, we identified a total of 60 fish taxa, with an average of 33.0 taxa per site. Of these, 20 taxa (33.3%) were non-native, distributed across 11 orders, 13 families, and 17 genera. We observed geographical homogenization among the native fish species, whereas non-native taxa exhibited varied geographical distributions—some taxa were widely distributed across river basins while others were restricted to specific sites or basins. Simple linear regression analyses revealed positive correlations between the number of taxa and species richness for both native and non-native taxa. Co-occurrence network analysis indicated positive relationships among both native and non-native taxa, with only two negative relationships involving one native and two non-native fish taxa.

**Abstract:**

Rapid urbanization and its associated human activities have facilitated the colonization and spread of non-native species, rendering urban ecosystems, particularly in megacities such as Beijing, highly susceptible to biological invasions. This study employed environmental DNA (eDNA) metabarcoding to evaluate the biodiversity and geographical distribution of non-native fish, as well as their interactions with native fish species, across three river basins in Beijing pertaining to the Daqing River, the North Canal, and the Ji Canal. Across all the 67 sampling sites, we identified 60 fish taxa, representing 11 orders, 23 families, and 40 genera, with an average of 33.0 taxa per site. Of these, 40 taxa were native, accounting for only 47.1% of the historically recorded native fish species. Additionally, we detected 20 non-native fish taxa, spanning 11 orders, 13 families, and 17 genera. Native fish exhibited geographical homogenization across the basins, while non-native taxa displayed varied geographical distributions. Non-metric multidimensional scaling (NMDS) and analysis of similarities (ANOSIM) revealed no significant variation in the non-native communities across the river basins. Although most of the non-native taxa were widespread, some were restricted to specific sites or basins. The North Canal exhibited significantly lower non-native biodiversity compared with the Ji Canal across all alpha diversity indices. Simple linear regression analyses indicated positive correlations between the number of taxa and species richness for both native and non-native taxa. Interestingly, species co-occurrence analyses revealed predominantly positive interactions among both native and non-native species pairs, with only two negative relationships involving one native and two non-native taxa. This study provides insights into the biodiversity and geographical distribution of non-native fish in Beijing and establishes a baseline for future biomonitoring and conservation efforts. The findings underscore the need for further investigation into the mechanisms and dynamics of biological invasions within urban environments in Beijing.

## 1. Introduction

Rapid urbanization and increasing human activities have significantly enhanced opportunities for invasive species to colonize and spread, making urban ecosystems hotspots for biological invasions [1,2]. Aquatic ecosystems within urban areas are particularly susceptible, as invasions are facilitated by the availability of multiple pathways and human modifications that favor invaders [1,3]. Urban aquatic environments, which are central to commerce, aquaculture, pet trade, and food sales, often receive non-native species either accidentally or intentionally [4]. Moreover, the practice of “prayer animal release”, a form of compassionate animal liberation, is prevalent in many cities, leading to the establishment of non-native species in local habitats [5]. Water diversion projects that transfer water between river basins to meet urban demands can create “invasion highways” [6], bypassing natural barriers and unintentionally introducing invasive species from source to recipient basins [6,7,8]. Compared with traditional pathways, these diversion-associated highways can rapidly and extensively introduce an array of non-native species into local environments [6]. Furthermore, human activities such as recreational use and ecological restoration can facilitate the spread of these introduced species across urban water bodies, advancing invasion fronts in urban aquatic ecosystems [7,9].

In addition to multiple invasion pathways, urbanization significantly alters ecological conditions in ways that favor non-native species [1,10,11]. Urban water bodies typically have higher levels of pollutants [5,12]. These pollutants can degrade aquatic ecosystems, making them less hospitable for native species but more suitable for invasive species that can tolerate or even benefit from these changes [13]. Pollution and other disturbances from human activities often simplify the structure and alter the functioning of aquatic ecosystems, leading to the loss of native competitors and predators [14]. This decline in biotic resistance allows non-native species to establish and proliferate more easily [15]. Moreover, studies have shown that urbanization acts as a strong selective force, favoring traits such as rapid growth and high fecundity in non-native species, enhancing their ability to survive and reproduce in urban environments [16]. Combined with the high availability of multiple invasion pathways, these factors create urban aquatic environments where non-native species can thrive, leading to biological invasions that disrupt native ecosystems and cause various ecological and economic problems [1,3,15,17]. Thus, integrated management and prevention strategies are essential to mitigate the impacts of invasive species in urban aquatic ecosystems. This is especially critical in megacities, where escalating human activities and multiple invasion pathways promote the colonization and spread of non-native aquatic species, thereby accelerating their ecological impacts [3,17].

Beijing, the capital of China and a historically significant megacity in Northern China, has experienced substantial urban expansion over the past century. Its population has increased nearly fivefold over the last 70 years, reaching 21.86 million, according to the 2023 census, while its area has expanded to 16,410 km^2^. This rapid urbanization has resulted in the creation of a complex network of man-made, semi-natural, and natural lentic and lotic ecosystems, which are divided into five river basins pertaining to the following areas: the Ji Canal, the North Canal, the Daqing River, the Chaobai River, and the Yongding River. However, these basins have been significantly influenced by various activities, including flood control construction, park development, transportation infrastructure, ecological restoration, water diversion, trade, and commerce [3,6,8]. These human activities have had both direct and indirect adverse effects on the city’s biodiversity [3,18]. Despite an initial record of high fish diversity, including 12 orders, 21 families, 65 genera, and 85 species, there has been an approximately 50% decline in native species richness and significant reductions in their distribution ranges [18]. A recent study employing environmental DNA (eDNA) techniques has identified 23 non-native fish taxa, accounting for 30.7% of the total fish biodiversity detected, with many of these taxa being highly invasive and having considerable ecological impacts [3]. These findings highlight the critical need for enhanced management strategies to monitor and conserve urban fish biodiversity, particularly through advanced techniques such as eDNA metabarcoding.

After collecting eDNA shed by fish into their habitats, the application of fish universal PCR primers combined with next-generation sequencing (i.e., eDNA metabarcoding) enables researchers to rapidly and comprehensively assess the biodiversity of the entire fish community [19,20]. Consequently, eDNA metabarcoding has proven to be both efficient and cost-effective, requiring less expertise compared with traditional morphological identification methods [3,19]. Studies have demonstrated that eDNA metabarcoding exhibits high sensitivity, allowing for the detection of a broad spectrum of rare taxa and those difficult to capture with traditional survey methods [20,21,22]. This advantage is crucial for detecting non-native invasive species, as their populations often remain extremely small for extended periods before becoming invasive (i.e., lag time) [9,23].

Given the rapid decline in fish biodiversity and the increasing proportion of non-native fish taxa across river basins in Beijing, we hypothesize that the introduction and subsequent spread of non-native fish have negatively impacted native fish taxa. To test this hypothesis, we used eDNA metabarcoding on samples collected from three river basin areas in Beijing—Daqing River, North Canal, and Ji Canal—each of which has been significantly disturbed by human activities. Our objectives were to assess the biodiversity and geographical distribution of non-native fish and to examine their interactions with native fish taxa in these urban river systems.

## 2. Materials and Methods

### 2.1. Sampling

**Figure 1:**
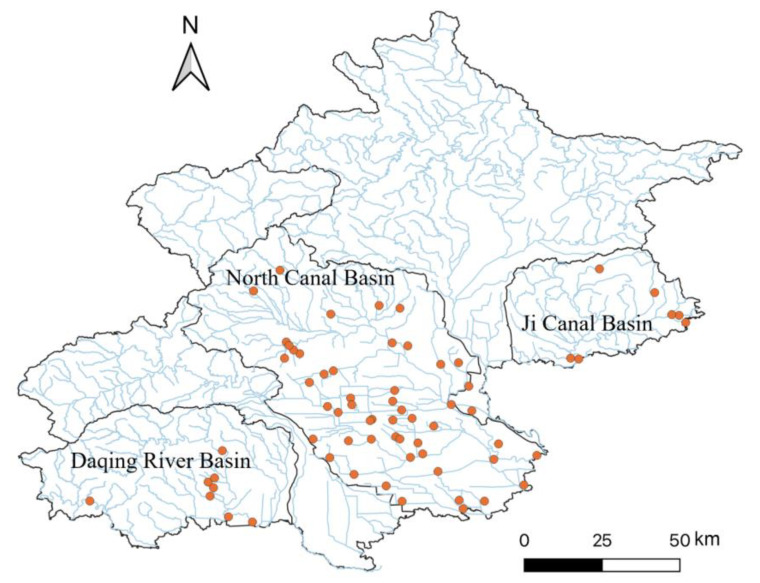
Sampling sites across three river basins in Beijing.

Based on a previous study [3] showing that urbanization, particularly factors related to water quality associated with human activities, significantly affects fish biodiversity in Beijing, this study focused on the North Canal Basin, situated in the city center (Figure 1). This basin has been notably affected by increased human activities, resulting in alterations in the water chemistry, riverbank, and bottom structures, and subsequent changes in the biotic community composition and functioning [5,12,24]. In addition to the North Canal Basin, we also sampled the Daqing River and Ji Canal Basins (see Figure 1). Overall, we conducted sampling at 67 sites (Table S1) across these three basins: 7 sites in the Ji Canal Basin, 8 sites in the Daqing River Basin, and 52 sites in the North Canal Basin (Figure 1).

For the lotic sites, where all the surveyed rivers are shallow (<1 m deep), we collected 1 L of water from 0.5 m below the surface at each site [5]. For the lentic sites, we collected a combined total of 2 L, consisting of two 1 L samples: one from 0.5 m below the surface and the other from 0.5 m above the bottom [5]. Triplicate samples were collected from both lotic and lentic sites, resulting in a total of 3 L of water for lotic sites and 6 L for lentic sites [5,7]. All samples were collected in sterile, nucleic acid-free bottles, stored at 4 °C, and transported to the laboratory. In the laboratory, the water samples were filtered through 0.45 µm mixed cellulose acetate membranes [7]. Additionally, sterilized ultrapure water was filtered as a negative control for each site [5,7]. The filtered membranes and negative controls were placed in centrifuge tubes, rapidly frozen in liquid nitrogen, and stored at −80 °C until eDNA extraction.

### 2.2. eDNA Purification, PCR Amplification, and Sequencing

For each sample, eDNA was extracted from the stored membranes using the CTAB (cetyltrimethylammonium bromide)-PCI (phenol–chloroform–isoamyl alcohol) method. This approach was chosen because it effectively removes PCR inhibitors, facilitating the subsequent metabarcoding analysis [25]. The concentration and quality of the extracted eDNA were evaluated with an ultraviolet spectrophotometer (NanoDrop, Thermo Scientific Inc., Wilmington, DE, USA) and by 2% agarose gel electrophoresis. The eDNA was then subjected to PCR amplification using fish-specific primers targeting the 12S mitochondrial rRNA gene: forward primer 5′-ACACCGCCCGTCACTCT-3′ and reverse primer 5′-CTTCCGGTACACTTACCATG-3′ [26,27]. Unique tag sequences were used for each site to distinguish individual samples. PCR amplification was conducted in a total volume of 25 μL, comprising 2.5 μL of 10× PCR Buffer (Takara, Kyoto, Japan), 2 μL of dNTPs (each 2.5 mM), 1.5 μL of each primer (10 μM), 2 U of *Taq* DNA polymerase (Takara), and 1 μL of the extracted eDNA. The amplification protocol included an initial denaturation at 95 °C for 5 min, followed by 35 cycles of denaturation at 95 °C for 30 s, annealing at 50 °C for 30 s, extension at 72 °C for 30 s, and a final extension at 72 °C for 10 min. The resulting PCR products were purified using the SanPrep column PCR product purification kit (Sangon Biotech, Shanghai, China). Equimolar amounts of purified PCR products from each site were combined to create a sequencing library, which was subsequently sequenced on the Illumina Novaseq 6000 platform (paired-end, 2 × 150 bp).

### 2.3. Bioinformatic Analysis

We followed the bioinformatics methods outlined in a previous study [28]. In brief, all sequences were processed using the Galaxy Platform (https://mem.rcees.ac.cn) and sorted into distinct files based on unique primer tags for each site. Sequences with no mismatches in their tags and, at most, two mismatches in each primer were retained for downstream analyses. Paired-end reads were merged with at least 50 bp of overlap, and tags and primers were removed from the merged sequences. Low-quality sequences containing undetermined nucleotides or quality scores below 20 (i.e., Phred *Q* < 20) were discarded. All cleaned sequences were clustered into Zero-radius Operational Taxonomic Units (zOTUs). The representative sequence of each zOTU was annotated against the GenBank nucleotide database using the BLASTN program integrated into the SEED 2 platform. Fish taxonomic annotation was performed using the methods described by Zhang et al. [3].

### 2.4. Data Analysis

We calculated several indices of alpha diversity, including the number of taxa, taxa richness, Simpson’s Index, and Pielou’s evenness, for both native and non-native fish. The relative abundance of each detected fish was determined by dividing the number of sequences obtained for a specific taxon by the total number of sequences for all fish taxa in each sample. To assess geographical patterns of native and non-native fish taxa among the river basins, we performed pairwise comparisons using the Mann–Whitney *U* test and visualized the results with box plots. Non-metric multidimensional scaling (NMDS) and analysis of similarities (ANOSIM) were conducted to evaluate variations in community composition of both the native and non-native fish among the river basins. To evaluate potential interactions between the native and non-native fish communities, we conducted simple linear regression analyses on diversity indices across all the sampling sites for both community types. Further, to assess interactions between each non-native and native fish taxa, we reconstructed co-occurrence networks based on all the fish taxa. Spearman correlations were calculated, and significant correlations with absolute r values above 0.5 and *p* values below 0.05 were included in the co-occurrence networks. False discovery rate (FDR) correction was applied to adjust *p* values for multiple comparisons. Relationships between non-native and native fish taxa were retained and visualized using Gephi 0.9.2 [29]. Alpha diversity indices, NMDS, and ANOSIM analyses were conducted using PRIMER v6 [30]. Network analysis was performed with the R package igraph [31].

## 3. Results

### 3.1. Fish Diversity

Following high-throughput sequencing, a total of 25,057,493 raw reads were generated from all 67 samples. After stringent quality control, 2,409,924 clean reads were retained for subsequent analyses, resulting in a total of 179 zOTUs. Taxonomic assignment revealed a relatively high level of biodiversity, with 60 identified taxa—31 taxa (51.7%) classified at the species level, 26 taxa (43.3%) at the genus level, and 3 taxa (5.0%) at the family level. These 60 taxa were further categorized into 11 orders, 23 families, and 40 genera (Table S2). The most abundant order was Cypriniformes, comprising 33 taxa (55.0%), followed by Gobiiformes (6 taxa; 10.0%), Anabantiformes (4 taxa; 6.7%), Cichliformes (4 taxa; 6.7%), and Siluriformes (4 taxa; 6.7%). The taxa-level rarefaction curves demonstrated that our sampling effort adequately captured the fish biodiversity at each sampling site (Figure S1).

The number of taxa detected across all 67 sampling sites varied widely, ranging from 17 taxa at site SX8 in the North Canal to 44 taxa at site S109 in the North Canal and site S92 in the Ji Canal, with an average of 33.0 taxa per site (Table 1 and Table S2). Similarly, the relative abundance of each species also varied significantly (Table S2). For example, the relative abundance of *Acheilognathus* sp. reached 97.90% at site S179 in the North Canal, while at some sites, such as S91 in the Ji Canal, its abundance was less than half of this value (Table S2).

After categorizing all the taxa into native and non-native groups, we identified 40 native taxa (66.7%) and a relatively large proportion of non-native taxa, totaling 20 (33.3%; Table 1 and Table S2). These non-native taxa were distributed across 8 of the 11 detected orders (72.7%), with 3 orders being exclusively represented by non-native taxa (Table S2). Non-native fish were also detected in 13 of the 23 observed families (56.5%), with 6 families being exclusively non-native. Additionally, 17 of the 40 identified genera (42.5%) included non-native taxa, with 14 genera being exclusive to non-native species (Table S2). Similarly to the native taxa, the most abundant order among the non-native taxa was Cypriniformes, comprising six taxa (30.0%). The second most abundant order was Cichliformes, which included four taxa and was exclusively represented by non-native species (Table S2). Among these 20 non-native taxa, 13 (65.0%) were classified at the species level, 6 (30.0%) at the genus level, and 1 (5.0%) at the family level (Table S2). Of the 13 taxa identified at the species level, 10 (76.9%) have documented invasion histories (Table 2). All these non-native fish were recorded in Beijing only recently, with records dating back to 2018 (Table 2). Notably, seven species were first detected in this study (Table 2).

The number of both native and non-native fish taxa varied among the sites, ranging from 16 to 36 with an average of 26.6 taxa per site for native species, and from 1 to 16 with an average of 8.0 taxa per site for non-native species (Table 1). Similarly to the overall number of taxa observed, the species richness of the non-native taxa was lower compared with that of the native taxa, ranging from 1.51 to 3.23 (average = 2.42) for native species versus 0 to 2.27 (average = 1.34) for non-native species (Table 1). However, both Simpson’s Index and Pielou’s evenness were higher for the non-native taxa, with Simpson’s Index ranging from 0 to 0.91 (average = 0.72) compared with 0.04 to 0.87 (average = 0.59) for the native taxa, and Pielou’s evenness ranging from 0 to 0.93 (average = 0.78) versus 0.05 to 0.72 (average = 0.45) for the native taxa (Table 1). Higher values of these indices indicate a more even distribution of the relative abundance of non-native taxa at each sampling site. Interestingly, the relative abundance of most non-native taxa was low, with average values below 0.2% across nearly all sampling sites (Table 3). However, a few non-native taxa exhibited relatively high abundances, such as the dark sleeper *Odontobutis potamophila*, reaching up to 8.73% at several sites in the North Canal (Table 3 and Table S2).

### 3.2. Geographical Distribution of Fish Diversity

**Figure 2:**
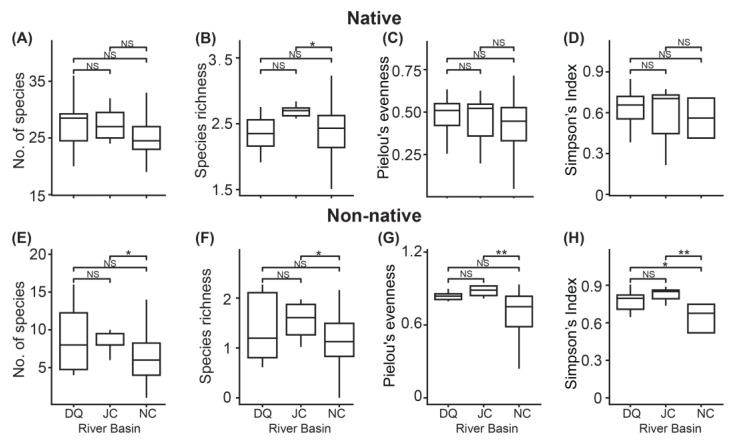
Comparison of alpha diversity indices among the river basins for fish communities detected by eDNA metabarcoding. DQ = Daqing River; JC = Ji Canal; NC = North Canal; NS = not significant in statistical analysis; *: *p* < 0.05; **: *p* < 0.01. (**A**) and (**E**): number of species for native and non-native fish, respectively; (**B**) and (**F**): species richness for native and non-native fish, respectively; (**C**) and (**G**): Pielou’s evenness for native and non-native fish, respectively; (**D**) and (**H**): Simpson’s index for native and non-native fish, respectively.

**Figure 3:**
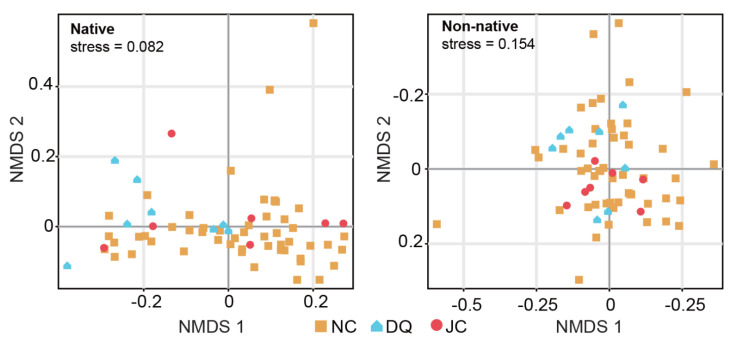
Non-metric multidimensional scaling (NMDS) analysis for both native and non-native fish taxa detected by eDNA-metabarcoding across the three river basins. DQ = Daqing River; JC = Ji Canal; NC = North Canal.

When grouping both native and non-native fish taxa by their basin origins, we found no significant differences in all the alpha diversity indices among the basins for the native taxa (Figure 2A–D), except for one pairwise comparison: species richness was significantly higher in the Ji Canal compared with the North Canal (*p* < 0.05; Figure 2B). For non-native taxa, we observed higher biodiversity in the Ji Canal compared with the North Canal (Figure 2E–H), with a greater number of taxa (*p* < 0.05), higher species richness (*p* < 0.05), greater Pielou’s evenness (*p* < 0.01), and a larger Simpson’s Index (*p* < 0.01). Additionally, the Simpson’s Index was also larger (*p* < 0.05) in the Daqing River compared with the North Canal. Further NMDS and ANOSIM analyses revealed no significant community variations in both native and non-native fish taxa among the basins (*p* > 0.05; Figure 3).

Upon closer examination of the positive detections of non-native taxa among the sampling sites, the percentage of positive detections for most species was generally similar (Table 3). However, substantial differences were observed for several species. For example, *Acheilognathus rhombeus* was detected at over 40% of the sampling sites in the Ji Canal Basin, while it was found at only 7.7% of sites in the North Canal Basin, despite extensive sampling efforts (Table 3). Similarly, *Centrarchidae* sp. was detected at 37.5% of sites in the Daqing River Basin but only at 9.6% of sites in the North Canal Basin (Table 3). We identified three basin-specific non-native taxa: *Channa striata* and *Gambusia affinis* in the Daqing River Basin and *Lepomis* sp. in the North Canal Basin. These taxa were also site-specific and exhibited relatively low abundances (Table 3). These patterns indicate a spatial distribution of these non-native fish, likely reflecting points of entry during their introduction.

### 3.3. Interactions among Fish Taxa

**Figure 4:**
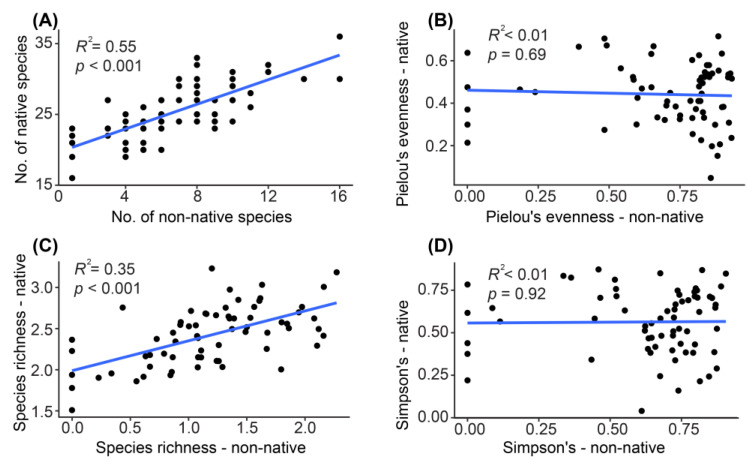
Simple linear regression analysis of four alpha diversity indices between the native and non-native taxa. (**A**): number of non-native species; (**B**): Pielou’s evenness for non-native species; (**C**): Species richness for non-native species; (**D**): Simpson’s index for non-native species.

**Figure 5:**
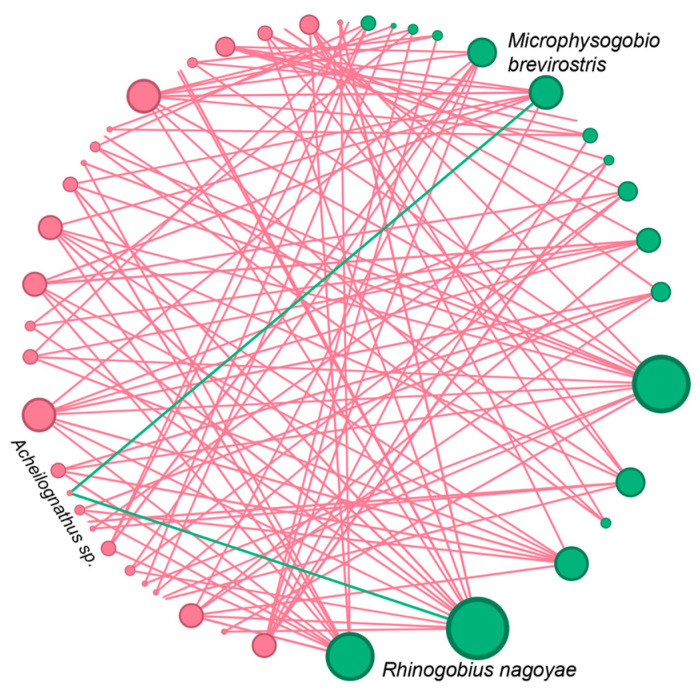
Species co-occurrence network reconstructed for both the native (pink circles) and non-native fish (green circles). The size of circle for each taxon represents its relative abundance across all the sampling sites. The pink and green lines show positive and negative relationships among species pairs, respectively. Species names are only shown for negative relationships.

Contrary to expectations, simple linear regression analyses of the alpha diversity between the non-native and native taxa did not reveal any significant negative relationships (Figure 4). Instead, we observed significant positive relationships in the number of species and species richness between the native and non-native taxa (Figure 4). To investigate potential interactions between each non-native and native fish taxon more comprehensively, we constructed a co-occurrence network that included all the fish taxa. Interestingly, we detected positive relationships among almost all species, regardless of whether they were native or non-native (Figure 5). We identified only two negative relationship pairs involving one native and two non-native fish taxa: between *Rhinogobius nagoyae* and *Acheilognathus* sp. and between *Microphysogobio brevirostris* and *Acheilognathus* sp. (Figure 5).

## 4. Discussion

Biological invasion and urbanization are closely interconnected processes, with various anthropogenic activities in urban environments significantly facilitating the entire invasion process, including the introduction, establishment, and spread of non-native species [1,2,10,11,12]. Consequently, a large number of non-native species can rapidly colonize and spread in urban ecosystems, outcompeting native species and altering the ecological balance [16,32]. Consistent with observations in aquatic ecosystems of many megacities, we found a high proportion of non-native fish in Beijing, accounting for 33.3% of all the detected fish taxa (Table 1 and Table S2). Despite using a highly sensitive eDNA-based method, only 40 native fish taxa were detected, which is considerably fewer than the historically documented native fish biodiversity in Beijing, which comprises 85 species across 12 orders, 21 families, and 65 genera [18]. While no significant geographical differences were detected among the river basins for native fish, the North Canal Basin exhibited lower non-native fish biodiversity compared with the Ji Canal Basin, despite more intensive sampling efforts (Figure 2). We observed positive relationships among the taxa at both the community (Figure 4) and species levels (Figure 5), with only two instances of negative relationships.

### 4.1. Monitoring Diverse Non-Native Fish

Using eDNA metabarcoding, we detected a substantial number of non-native fish taxa in Beijing, with 20 taxa identified, representing 33.3% of the total fish diversity (Table 1 and Table S2). The high prevalence of non-native species in urban aquatic ecosystems is primarily due to multiple introduction pathways associated with human activities [3,10,33]. However, pinpointing specific introduction pathways is challenging due to the complex nature of these pathways and the interactions among various human activities [34,35]. Additionally, many non-native species exhibit a prolonged lag phase during which their populations remain low before becoming invasive [36]. Consequently, tracking the causes of invasions can be difficult, as much of the evidence needed for pathway analysis is often unavailable. In this study, we encountered similar challenges in identifying definitive pathways for the non-native fish taxa. For instance, the South-to-North Water Transfer Project (SNWTP), which channels water from the Yangtze River Basin to Beijing, has likely facilitated the spread of non-native species by establishing an “invasion highway” [6,7,8]. The dark sleeper (*Odontobutis potamophila*), native to the Yangtze River Basin, appears to have used this pathway to invade Beijing. Although direct evidence is lacking, its common detection along the SNWTP channel [37] suggests that this water diversion was a potential pathway for its introduction and spread. Water diversion projects, such as the SNWTP, continuously transport large volumes of water and propagules, which significantly increases the likelihood of successful invasions [38,39]. This may explain the high relative abundance of the dark sleeper observed across the three river basins and particularly in the North Canal Basin, where the diverted water is initially released (Table 3). Similarly to other species introduced by this project, such as the golden mussel (*Limnoperna fortunei*) [6,7,8], the large volume of propagules can enhance rapid spreading and potential ecological impacts [7]. While this study has not yet documented significant negative effects caused by the dark sleeper, this may be due to the invasion being in its early stages. Field surveys have identified large populations at sites where high eDNA abundance was detected (personal observations). Given the dark sleeper’s predatory nature and documented invasion in other regions, such as the Edo River in Japan, it is crucial to increase monitoring and eradication efforts for this and other non-native species detected in Beijing.

Interestingly, the relative abundance of some non-native fish species was low (Table 3 and Table S2), suggesting that these species are likely in the early stages of invasion at the surveyed sites. The high sensitivity of eDNA metabarcoding enabled the detection of these non-native species even at very low population densities [7,40,41]. Early detection is crucial for the effective management of newly introduced species, as it allows for timely responses and the implementation of eradication and control measures [7,42]. With 10 out of 13 identified non-native species having documented invasion histories (Table 2), the early detection in this study provides a valuable baseline for monitoring their population dynamics and tracking their spread across water bodies in Beijing.

The early detection of low-abundance non-native fish in Beijing presents several technical challenges, including both false negatives (Type II errors) and false positives (Type I errors) [43,44]. False negatives for low-abundance taxa often arise from stochastic effects or random sampling errors inherent in the eDNA metabarcoding process [45,46]. These errors can occur at various stages, including during the eDNA sampling, PCR amplification, and sequencing [45,46,47]. The cumulative effect of these random sampling errors can significantly reduce the detection probability of rare non-native species, leading to higher rates of false negatives. To mitigate this, monitoring baselines should be based on the union of non-native taxa identified across multiple studies or surveys rather than on their intersection. Additionally, targeted methods, such as combining passive and active eDNA sampling strategies and using species-specific primers, can enhance the detection of rare species and improve the accuracy of monitoring and eradication efforts [48,49,50].

Due to limited natural water resources in Beijing, rivers are supplemented with unconventional sources, such as treated wastewater from various human activities [20,51]. The discharge of treated wastewater introduces eDNA into natural water bodies, raising concerns about false positives [20]. Despite advanced treatment strategies, a diverse array of fish species can still be detected in the treated effluent [20]. This issue of eDNA pollution may influence the results of this study, particularly for newly detected non-native fish used for food, such as *Coptodon zillii*, and those reared as pets, such as *Microphysogobio brevirostris* (Table 2). While eDNA detection indicates the presence of these species in the sampled environment, it does not confirm the presence of live individuals. This limitation of eDNA-based methods underscores that positive detection does not always align with direct observations or physical evidence. While we cannot completely eliminate the potential errors caused by eDNA pollution, we ensured that the water samples were collected upstream or away from wastewater treatment plants. Additionally, the presence of non-commercial species not valued for food, aquaculture, or pets (Table 2), along with their capture or observation at sites (personal observations and multiple detections in references [3,18]), supports their presence in Beijing. To address false positives from eDNA pollution, using eRNA instead of eDNA for routine monitoring could be considered, as eRNA generally has faster decay rates in aquatic environments [52]. Complementary methods such as field surveys should also be conducted to verify the presence of taxa detected by eDNA-based methods. Moreover, relying solely on public libraries for BLAST searches can lead to false positives in taxonomic annotation. In this study, after annotating against GenBank, most of the fish taxa detected had either been observed in field surveys (historical data [18]) or identified in other eDNA-based studies [3]. However, this cross-validation does not completely rule out the possibility of false positives. To mitigate this limitation, constructing local reference libraries for taxa is essential for accurate taxonomic annotation [53,54]. We have begun building reference libraries for fish and other taxa in Beijing, though species enrichment, particularly for rare taxa, remains a challenge due to difficulties in physically capturing these species in large water bodies. Using a combination of local and public libraries can enhance the efficiency and accuracy of taxonomic annotation.

### 4.2. Geographical Distribution of Fish in Beijing

The current geographical distribution of fish fauna reflects long-term, complex interactions among geological, evolutionary, and environmental processes [55,56,57]. However, urbanization can rapidly alter the community structures and geographical distributions of freshwater fish, leading to native species loss and the introduction of non-native species [58,59]. This often results in the geographical homogenization of flora and fauna in urban ecosystems, eroding evolutionary signals and geographical isolation in biological communities [60,61,62]. Consistent with numerous studies, we observed no differences in biodiversity for the native fish taxa across all indices except for species richness between the Ji Canal and North Canal (Figure 2), no site-specific or even basin-specific taxa (Table S2), and no detectable significant variations in community geographical distributions among the three surveyed basins in Beijing (Figure 3). Many factors associated with anthropogenic changes significantly contribute to homogenization. These include abiotic factors, such as environmental pollution, water disconnection, loss of habitat diversity and riparian vegetation due to modifications of riverbanks and bottoms in hydraulic engineering projects, as well as biotic factors such as the introduction of invasive species [3,59,60,63]. These influential factors often lead to large-scale extinctions, particularly affecting species that are sensitive to environmental changes and those that are endangered or endemic to specific/unique habitats [59,64]. The remaining widespread or highly tolerant species contribute to the high level of geographical homogenization within a river basin. Indeed, comparing historical records of 85 native fish species with the detection of 40 native species in this study clearly indicates that more than half of the native species have been extirpated. It should be noted that molecular methods are more sensitive in biodiversity detection and allow easier coverage of larger geographical scales than traditional surveys, which recorded 85 native fish species at a time when molecular methods were unavailable. Thus, some rare or endemic taxa could have been missed by those traditional surveys, leading to an extinction rate much higher than that observed here. Additionally, we observed a relatively large proportion of low-abundance native taxa in this study (Table S2), further suggesting that extinction will likely accelerate rather than slow down if no further actions are taken. Consequently, the detected low-abundance species in this study provide a valuable baseline for future monitoring in conservation programs.

Interestingly, in contrast to the high level of geographical homogenization observed in native fish, we observed geographical distribution patterns for the non-native fish; while most taxa showed a widespread distribution across the three river basins, some taxa were site- and basin-specific (Table 3). The site-specific taxa, along with their low abundance at the site, suggests that the positive sites might represent the initial points of invasion into Beijing. When considering the whole basin, we detected higher biodiversity in the Ji Canal compared with the North Canal across all indices analyzed (Figure 2). As these two basins are situated in regions with varying levels of urbanization and human activities, different invasion pathways derived from varied human activities may introduce diverse pools of propagules. Additionally, the interactions between the local environment and the introduced propagules may contribute to the varied establishment of non-native fish in local waters [65,66].

### 4.3. Interactions among Fish Taxa

Indeed, the extinction of native species due to urbanization is just one of many factors contributing to geographical homogenization in urban aquatic ecosystems. The introduction of non-native species is a major driver of homogenization, which is achieved through numerous mechanisms such as competitive exclusion [67], predation [68], habitat alteration [69], and the disruption of ecological processes [70]. Contrary to expectations of observing significantly negative relationships between native and non-native fish taxa, we found only limited effects of non-native fish on native taxa, with just two negative relationships detected (Figure 4 and Figure 5). Despite significant efforts in ecological restoration and pollution control for both aquatic and terrestrial ecosystems in Beijing [71,72], water pollution, particularly from the discharge of large volumes of treated wastewater resulting from large populations, continues to threaten biodiversity [73,74]. Harsh environments also pose a threat to non-native taxa, especially those recently introduced with low population densities [75,76]. More importantly, local environmental stress can largely alter the relationships and interactions between native and non-native taxa [77]. For example, a study showed that non-native species experienced facilitation by native communities in stressful regions and competition with native communities in regions with lower environmental stresses [77]. Meanwhile, the author proposed a positive relationship between non-native invaders and native biodiversity in stressful habitats [77]. Our findings support this speculation, as we detected predominantly positive relationships between native and non-native fish taxa in the species co-occurrence network reconstruction (Figure 5). Further detailed investigations into the positive versus negative effects, as well as their transition across different environments, are needed to elucidate the mechanisms of invasion dynamics in urban environments with varying levels of stress.

## 5. Conclusions

Using eDNA metabarcoding, we conducted a comprehensive survey of fish diversity across various sampling sites in three river basins in Beijing. Our analysis identified a total of 60 fish taxa, representing 11 orders, 23 families, and 40 genera, with over one-third of these taxa being non-native. We observed geographical homogenization among the native fish across the river basins, while the non-native taxa exhibited variable geographical distributions. Although most of the non-native taxa were present in multiple basins, some were restricted to specific sites or basins. The non-native taxa displayed greater evenness in their distribution but were generally less abundant compared with the native species. The spatial patterns of the non-native taxa suggest recent introductions and localized establishment. Notably, interactions between the native and non-native species were predominantly positive, indicating a complex mechanism of species coexistence within the studied ecosystems. These findings highlight the dynamic nature of fish communities in urban environments and reveal intricate relationships between native and non-native species. The study provides valuable insights into the biodiversity and geographical distribution of non-native fish in Beijing and establishes a baseline for future biomonitoring and conservation efforts. Additionally, our results here emphasize the need for further research into the mechanisms and dynamics of species invasions in urban settings, where frequent human activities significantly impact aquatic ecosystems and biotic interactions.

## Figures and Tables

**Table 1:** Alpha diversity indices calculated for all the 67 sampling sites across three river basins in Beijing based on the environmental DNA (eDNA) metabarcoding for fish. zOTU = zero-radius Operational Taxonomic Unit.

	Daqing River	North Canal	Ji Canal	All
No. of zOTUs (average)	54–117 (83.0)	37–117 (70.2)	69–114 (83.0)	37–117 (78.7)
No. of fish taxa (average)	17–44 (31.3)	24–39 (31.5)	31–44 (36.1)	17–44 (33.0)
No. of non-native fish taxa (average)	4–16 (9.0)	1–14 (6.4)	6–12 (8.7)	1–16 (8.0)
No. of native fish taxa (average)	20–36 (27.5)	16–33 (24.9)	24–32 (27.4)	16–36 (26.6)
Species richness—non-native (average)	0.61–2.27 (1.37)	0–2.16 (1.10)	1.02–1.97 (1.55)	0–2.27 (1.34)
Species richness—native (average)	1.91–3.19 (2.42)	1.51–3.23 (2.39)	2.03–2.84 (2.61)	1.51–3.23 (7.42)
Simpson’s Index—non-native (average)	0.45–0.91 (0.75)	0–0.87 (0.59)	0.74–0.89 (0.83)	0–0.91 (0.72)
Simpson’s Index—native (average)	0.38–0.85 (0.63)	0.04–0.87 (0.55)	0.21–0.77 (0.58)	0.04–0.87 (0.59)
Pielou’s evenness—non-native (average)	0.60–0.90 (0.81)	0–0.93 (0.66)	0.82–0.93 (0.88)	0–0.93 (0.78)
Pielou’s evenness—native (average)	0.25–0.63 (0.48)	0.05–0.72 (0.43)	0.20–0.63 (0.45)	0.05–0.72 (0.45)

**Table 2:** Non-native fish species identified across all 67 sampling sites based on environmental DNA (eDNA)-metabarcoding in Beijing (only species-level identifications are presented, as tracking native ranges and invasion histories at higher taxonomic levels is not feasible). Publications or databases documenting the invasion history of a specific species are labeled with “Y” (Yes), while those without trackable records are labeled with “N” (No). The “year of first detection” was determined based on all the available literature and regular surveys conducted over more than 10 years by the Beijing Hydrology Center.

Non-Native Species	Common Name	Native Range	Invasion History?	Year of First Detection
*Acheilognathus rhombeus*	Korean flat bittering	Japan, Republic of Korea	Y	2018
*Channa striata*	Striped snakehead	South and Southeast Asia	Y	This study
*Coptodon zillii*	Redbelly tilapia	Northern Africa, the Middle East	Y	This study
*Gambusia affinis*	Mosquito fish	North America	Y	This study
*Gobio gobio*	Common gudgeon	Central and temperate Eurasia	Y	2021
*Gobio soldatovi*	Soldatov’s gudgeon	Amur River, Sakhalin Island, Lake Buir	N	This study
*Hypomesus nipponensis*	Japanese smelt	Japan, Republic of Korea, Russia	Y	This study
*Ictalurus punctatus*	Channel catfish	North America, Mexico	Y	2018
*Microphysogobio brevirostris*	Short-nose gudgeon	Taiwan, China	N	This study
*Odontobutis potamophila*	Dark sleeper	South China, Vietnam	Y	2018
*Oreochromis niloticus*	Nile tilapia	Africa, the Levant	Y	2020
*Osteochilus salsburyi*	-	Laos, Vietnam, Southern China	N	This study
*Rhinogobius nagoyae*	Nagoya goby	Republic of Korea, Japan	Y	2018

**Table 3:** The relative abundance (Abund.) and average (Av.) of the 20 non-native fish taxa across all 67 sampling sites detected using environmental DNA (eDNA) metabarcoding in Beijing. det.? = whether a non-native taxon was detected (Y) or not (N) in each river basin; %det. = percentage of surveyed sites with positive detection in each river basin (the number of positive sites/the number of total sites in each river basin × 100%).

Species	Abund. % (Av.) %	Daqing River Basin		North Canal Basin		Ji Canal Basin	
		det.?/ %det.	Abund. (Av.) %	det.?/ %det.	Abund. % (Av.) %	det.?/ %det.	Abund. % (Av.) %
*Acheilognathus rhombeus*	<0.01–0.73 (0.20)	Y/12.5	0.14 (-)	Y/7.7	0.02–0.53 (0.21)	Y/42.9	<0.01–0.73 (0.37)
*Barbatula* sp.	0.01–0.65 (0.11)	Y/62.5	0.01–0.20 (0.09)	Y/71.2	0.01–0.65 (0.13)	Y/85.7	0.01–0.21 (0.10)
*Centrarchidae* sp.	0.01–0.14 (0.04)	Y/37.5	0.03–0.14 (0.07)	Y/9.6	0.01–0.07 (0.03)	N	-
*Channa striata*	0.04 (-)	Y/12.5	0.04–0.04 (0.04)	N	-	N	-
*Cichlidae* sp.	0.01–0.56 (0.11)	Y/100.0	0.01–0.22 (0.10)	Y/78.8	0.01–0.40 (0.10)	Y/85.7	0.03–0.56 (0.27)
*Clarias* sp.	0.01–0.09 (0.03)	Y/50.0	0.01–0.09 (0.03)	Y/25.0	0.01–0.09 (0.04)	Y/42.9	0.01–0.03 (0.02)
*Coptodon zillii*	<0.01–0.32 (0.08)	Y/87.5	<0.01–0.31 (0.07)	Y/69.2	0.01–0.32 (0.10)	Y/85.7	0.03–0.21 (0.10)
*Gambusia affinis*	<0.01 (-)	Y/12.5	<0.01 (-)	N	-	N	-
*Gobio gobio*	<0.01–0.10 (0.02)	Y/37.5	<0.01–0.02 (0.01)	Y/19.2	<0.01–0.10 (0.02)	Y/14.3	0.01–0.01 (0.01)
*Gobio soldatovi*	<0.01–0.45 (0.11)	Y/50.0	<0.01–0.14 (0.04)	Y/21.2	0.01–0.45 (0.14)	Y/42.9	0.01–0.30 (0.15)
*Gymnogobius* sp.	0.01–0.05 (0.03)	Y/12.5	0.04–0.04 (0.04)	Y/5.8	0.01–0.03 (0.02)	Y/14.3	0.05–0.05 (0.05)
*Hypomesus nipponensis*	<0.01–0.10 (0.04)	Y/25.0	<0.01–0.02 (0.01)	Y/7.7	<0.01–0.09 (0.04)	Y/14.3	0.10–0.10 (0.10)
*Ictalurus punctatus*	<0.01–0.78 (0.10)	Y/50.0	<0.01–0.31 (0.12)	Y/26.9	<0.01–0.78 (0.14)	Y/14.3	0.08–0.08 (0.08)
*Lepomis* sp.	0.18 (-)	N	-	Y/1.9	0.18 (-)	N	-
*Microphysogobio brevirostris*	0.01–0.04 (0.03)	Y/37.5	0.03–0.04 (0.04)	Y/3.8	0.01–0.03 (0.02)	Y/14.3	0.01–0.01 (0.01)
*Odontobutis potamophila*	<0.01–8.73 (0.47)	Y/75.0	<0.01–0.13 (0.07)	Y/69.2	0.01–8.73 (0.83)	Y/71.4	0.01–0.11 (0.06)
*Oreochromis niloticus*	<0.01–0.15 (0.03)	Y/25.0	0.04–0.06 (0.05)	Y/50.0	<0.01–0.15 (0.03)	Y/85.7	0.02–0.10 (0.07)
*Oreochromis* sp.	<0.01–0.26 (0.06)	Y/62.5	0.02–0.26 (0.09)	Y/59.6	<0.01–0.17 (0.06)	Y/85.7	0.02–0.06 (0.04)
*Osteochilus salsburyi*	<0.01–1.04 (0.11)	Y/25.0	<0.01–0.13 (0.07)	Y/44.2	<0.01–1.04 (0.16)	Y/71.4	0.03–0.21 (0.12)
*Rhinogobius nagoyae*	<0.01–1.21 (0.17)	Y/87.5	0.01–0.18 (0.09)	Y/65.4	<0.01–1.21 (0.23)	Y/100.0	0.01–0.21 (0.11)

## Data Availability

All data needed to evaluate the conclusions in this paper are presented in the text.

## Supplementary Materials

The following supporting information can be downloaded at: https://www.mdpi.com/article/10.3390/ani14172532/s1, Figure S1: Rarefaction curves of the detected fish taxa for each sampling site; Table S1: The site codes, longitudes, and latitudes for the 67 sampling sites in the three river basins - North Canal, Daqing River, and Ji Canal; Table S2: List of fish taxa with relative abundance at each sampling site.
