# Supplementary figures and images for: Environmental DNA-Based Identification of Non-Native Fish in Beijing: Diversity, Geographical Distribution, and Interactions with Native Taxa
Source: Animals (Basel). 2024 Aug 31;14(17):2532. doi: 10.3390/ani14172532 (PMC11393847; doi:10.3390/ani14172532)

Supplementary file

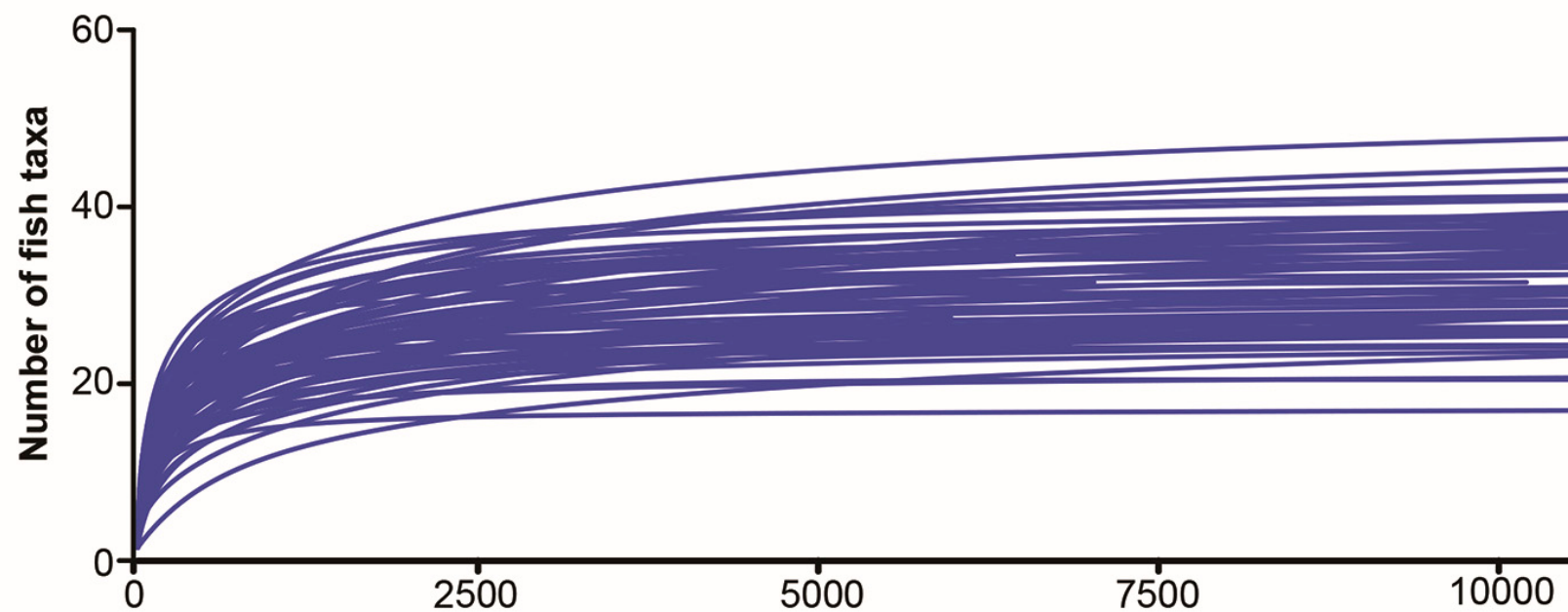

**Fig. S1** Rarefaction curves of the detected fish taxa for each sampling site.

Supplement: Supplementary file 1 [file animals-14-02532-s001.zip › Fig S1.pdf]
